# Effects of Semaglutide on Body Weight in Clozapine-Treated Individuals with Schizophrenia and Obesity: A Randomized Controlled Trial

**DOI:** 10.1192/j.eurpsy.2026.10638

**Published:** 2026-07-31

**Authors:** D. Siskind, M. Trott, U. Arnautovska

**Affiliations:** University of Queensland, Brisbane, Australia

## Abstract

**Introduction:**

People with schizophrenia have a markedly reduced life expectancy, largely due to cardiometabolic disease. Clozapine, the most effective antipsychotic for treatment-resistant schizophrenia, carries the highest risk of weight gain and metabolic dysfunction. Glucagon-like peptide-1 receptor agonists (GLP-1RAs), such as semaglutide, have demonstrated substantial weight loss in the general population, but their efficacy and safety in people with schizophrenia remain unknown.

**Objectives:**

This study aimed to evaluate the efficacy and safety of semaglutide for weight reduction in individuals with schizophrenia treated with clozapine.

**Methods:**

The COaST trial was a phase 2, multi-centre, randomised, placebo-controlled, participant and assessor-blinded trial conducted across six Australian sites. Adults (≥18 years) with schizophrenia or schizoaffective disorder, BMI ≥26 kg/m², and on clozapine for ≥18 weeks were randomised 1:1 to receive once-weekly subcutaneous semaglutide (titrated to 2.0 mg) or placebo for 36 weeks. The primary outcome was percentage body weight change. Secondary outcomes included BMI, metabolic parameters, psychosis symptoms (PANSS), and clozapine/norclozapine levels. Adverse events were systematically recorded.

**Results:**

A total of 31 participants were randomised (semaglutide n=15; placebo n=16). At 36 weeks, semaglutide resulted in a mean 13.88% (SE=0.90) reduction in body weight compared to 0.42% (SE=0.93) with placebo (between-group difference −13.46%; p<0.001). Additionally, 66.7% of semaglutide-treated participants achieved ≥10% weight loss versus 0% in placebo. There were no significant differences in clozapine/norclozapine levels or psychotic symptom severity. Semaglutide was generally well tolerated, with gastrointestinal adverse events (nausea, vomiting, diarrhoea) being the most reported but not leading to study withdrawal in most cases.

**Image 1:**
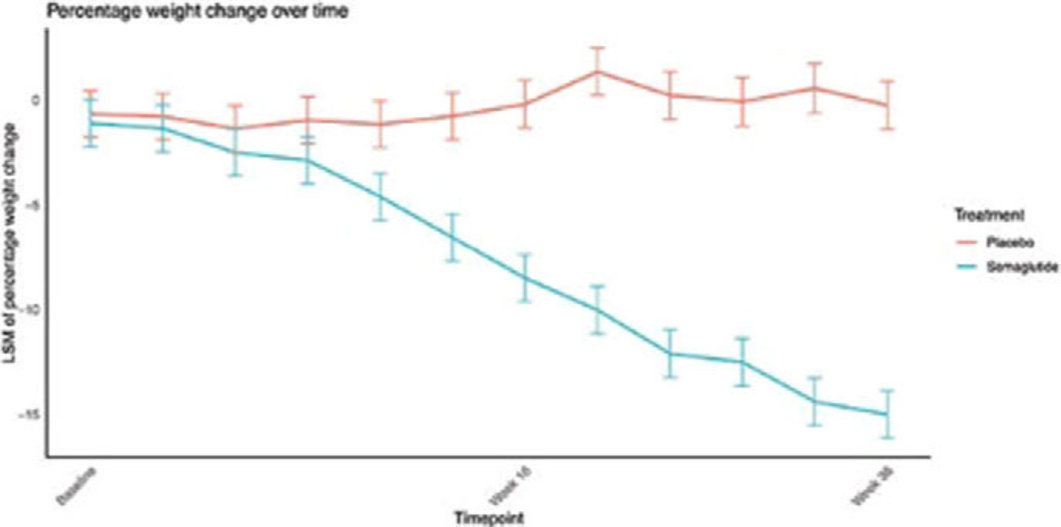
Image 1: Long description.

**Conclusions:**

In this small trial, semaglutide demonstrated substantial weight loss without impacting psychotic symptoms or clozapine levels. These findings provide preliminary evidence supporting semaglutide as a potential intervention for antipsychotic-associated obesity in schizophrenia and warrant larger trials to confirm efficacy and long-term safety.

**Disclosure of Interest:**

None Declared

## Long descriptions

### Image 1: Long description

The Y-axis is labeled L S M of percentage weight change, ranging from negative 15 to 0. The X-axis is labeled Timepoint, with markers for Baseline, Week 16, and Week 36. A legend on the right identifies two treatment groups: Placebo in orange and Semaglutide in teal.

* The Placebo group starts near 0 at Baseline and maintains a relatively flat trend, fluctuating slightly between negative 1 and positive 1 percent through Week 36.

* The Semaglutide group starts near negative 1 at Baseline and shows a steady, linear decrease in weight. By Week 16, the weight change is approximately negative 8 percent. The downward trend continues to Week 36, reaching approximately negative 15 percent.

* Both lines include vertical error bars at each timepoint, indicating variability in the data.

Navigate back to Image 1:.

